# Molecular characterization of pathogenic 4/91-like and QX-like infectious bronchitis virus infecting commercial poultry farms in Indonesia

**DOI:** 10.14202/vetworld.2019.277-287

**Published:** 2019-02-20

**Authors:** Michael H. Wibowo, Teridah E. Ginting, Widya Asmara

**Affiliations:** 1 Department of Microbiology, Faculty of Veterinary Medicine (FKH), Gadjah Mada University (UGM), Jl. Fauna No. 2, Karangmalang, Yogyakarta 55281, Indonesia; 2 Division of Immunology, Mochtar Riady Institute for Nanotechnology and Medical Science Group, University of Pelita Harapan. Jl. Boulevard Jend. Sudirman 1688, Lippo Karawaci, Tangerang, Banten 15811, Indonesia

**Keywords:** avian infectious bronchitis virus, Indonesia, nephropathogenic, QX strain, 4/91 strain

## Abstract

**Background and Aim:**

Existing data on the characteristics of infectious bronchitis virus (IBV) gathered throughout Indonesia have been recognized to indicate variants similar to globally distributed vaccine strains. Despite past and current intensive vaccination programs, IBV infections in the country’s poultry industry have not been effectively controlled. Therefore, this study aimed to investigate the genotype of several isolates based on partial S1 gene sequences. In particular, the investigation is directed to focus on layer chickens in actively vaccinated farms indicating IBV symptoms.

**Materials and Methods:**

Samples were isolated from ten different layer chicken flocks experiencing respiratory problem, drops in egg production, and a “penguin-like” stance, which were collected from commercial poultry farms in Central Java and Yogyakarta regions, Indonesia, within the periods of 2012-2018. Fragment of the S1 gene of IBV sampled from actively vaccinated commercial poultry farms was amplified using primer 5’-aca tgg taa ttt ttc aga tgg-3’ (forward) and 5’-cag att gct tac aac cac c-3’ (reverse) with the length of polymerase chain reaction (PCR) product at 383 bp. The sequence of samples was then compared with the sequence of reference S1 gene nucleotides of IBV from NCBI GenBank database. The amino acid analysis and multiple alignment sequence were conducted using Mega X.

**Results:**

During necropsy, enlargement of the oviduct and swollen kidney were observed. Reverse transcription-PCR diagnosis of their 383 bp S1 gene showed that all samples were IBV positive. Phylogenetic analysis of the S1 gene discovered seven samples to be clustered as 4/91-like strains. Meanwhile, the remaining three samples were grouped in QX-like strain cluster.

**Conclusion:**

This study is a pioneering report providing molecular evidence of pathogenic QX-like and 4/91-like strains circulating in Indonesia. Findings discovered, in this study, strongly suggested the importance of improving protections by available IBV vaccines through updated circulating strain clusters. It is critical to ensure the delivery of an effective control measurement of and vaccination protocols against IBV infections in the country’s commercial poultry industry in particular and worldwide in general.

## Introduction

Infectious bronchitis (IB) is recognized as a poultry-affecting contagious and acute respiratory disease caused by IB virus (IBV) [1]. The virus itself is an avian pathogen from the family *Coronaviridae*. IBV has been detected worldwide wherever poultry is reared, and in fact, the virus is highly potential to spread rapidly among non-protected poultries [2]. In infected chickens, IBV primarily affects the upper respiratory tract. As a matter of facts, high mortality in chickens is usually associated with nephritis. In the laying phase, reproductive tract of layer and breeder poultry is potential to get negatively affected, causing decreases in egg quality and production volumes [3].

As aforementioned, IBV belongs to family *Coronaviridae*, by which IBV is known as a single-stranded positive-sense RNA virus and has similar genome organizations as other Coronavirus. The length of IBV genome is about 27.6 kb, by which it represents codes for four major structural proteins: The spike (S) glycoprotein, small enveloped (E) protein, membrane (M) glycoprotein, and nucleocapsid (N) [4,5]. Genetically, the gene order of IBV is 5’-replicase-S-E-M-N-UTR-3’, wherein the UTR covers untranslated regions. Furthermore, replicase is a viral enzyme that is used during genome replication, while the following regions (S-E-M-N) are membrane-bound structural proteins (S, E, and M proteins) and an accessory protein (nucleoprotein) [6]. In general, the S glycoprotein of any Coronavirus, including IBV, is recognized as having a virion surface, a rod-shaped protein that is post-translationally cleaved into two subunits, S1 and S2 [7]. Besides, it contains four domains that are involved in anchoring the protein into the lipid bilayer of a host cell. In detail, the S glycoprotein consists of 1162 amino acid which was sliced into two subunits, including N-terminal S1 subunit (535 amino acids) and C-terminal S2 subunit (627 amino acids) [8]. According to Cavanagh [9], S glycoprotein has the most varied structure among other proteins in IBV gene. In particular, three hypervariable regions (HVRs), in which base pairs of nucleotides repeat, exist in the first 395-amino acid region of the S1 subunit. Hence, the S1 domain has the most variable regions and carries most of the virus neutralizing epitopes. Besides, it is primarily responsible for an attachment process to the receptor on host cells [10]. Changes in a few amino acids within the S1 protein would result in a change of serotype. Variation in the S1 protein is, hence, used as the basis for genotype and serotype classification [11-13]. On the other hand, the S2 domain is relatively conserved, mediating the fusion of virus and cell membrane during an entry stage [6,14,15]. In terms of its functions, therefore, the existence of S glycoprotein is imperative during virus attachment, fusion activation, and the release of viral genome into a cell. In short, S protein of Coronavirus is a critical determinant of its pathogenicity and host range.

In general, there are three clinical manifestations of an IBV infection, which include respiratory problems, reproductive disorders, and nephritis. Besides, factors that influence clinical manifestations are age, pathogenicity of the virus strain, and immunity [16]. Based on tissue tropism, there are two major IBV pathotypes: Respiratory and nephropathogenic. Most of classic IBV cases, including the Massachusetts (Mass) serotype, infects respiratory tract. On the other hand, nephropathogenic strains, which occur mostly in Asian and Middle Eastern countries, infect and damage kidneys that may later result in a significant mortality rate [1,17]. Furthermore, the pathogenicity of IBV strains varies greatly on oviduct, in which various strains are able to replicate in oviduct epithelial cells. In particular, the QX strain is one of the nephropathogenic strains with clinical symptoms of diarrhea, proventriculitis, and losses of body weight in chickens aged 25-70 days [18]. First isolated in China, QX variant is now predominant in Asia and Europe and has since been showing altered tissue tropism. It is currently associated with respiratory problems, nephritis, cystic oviduct, drops in egg production, and “false layers” syndrome [1,17,19-24].

In the early 1930s, the first case of IB recorded in history occurred in the United States [25]. In fact, some serotypes, including QX-like IBV, Mass strain from the United States, 4/91 (CR88) from the United Kingdom, and H120 strains from the Netherlands, are recognized as variants causing local and regional impacts with high potentials to spread far and wide to other countries [3]. In Indonesia, IBV was first reported in 1977 [26], by which multiple IBV isolates have been studied since then. IBV isolates gathered in Indonesia included I-37 and I-269, both of which were isolated by Darminto [27], while isolate I-14 was isolated by Indriani and Darminto [28]. According to Dharmayanti *et al*. [29], isolate I-37 was classified into Connecticut (Conn) serotype. In general, IBV isolates from Indonesia are characterized as variants similar to globally distributed vaccine strains, including Australia-origin N2/62 and US-origin Conn 46 as well as Mass 41 [30,31].

Practically, vaccination is the most common method to prevent IBV infections. To do so, controlling IBV in commercial poultry farms is typically conducted by the application of live attenuated vaccines (LAV) and killed/inactivated vaccines. In Indonesia, IB vaccination commonly applies vaccines fitted to serotypes M41, H120, or Conn. Besides, several vaccine manufacturers use local IB virus gathered in the country [30]. However, IB disease continues to act as an active threat to poultry despite progressive vaccination programs. It is particularly caused by the continuous emergence of new IBV variants from time to time. In the period 2012-2018, a pilot study of this work has gathered samples from layer chicken farms/flocks in Central Java and Yogyakarta (Indonesia) experiencing signs of IBV infection such as respiratory problems, drops in production, “penguin-like” stance, cystic oviduct, and nephritis at necropsy. In recent years, there is a growing tendency in some of the country’s layer farms to report signs of QX-like infections; however, there is no scientific report that clearly proves and confirms the genotype of the agent based on molecular analysis.

Those many reports by poultry farmers on clinical manifestations in various IB cases that theoretically refer to QX-like infection, therefore, require a proper scientific proof at molecular level on the genotype of virus isolates found on the fields. To address the problem, this study aims to perform a phylogenetic analysis of S1 gene to reveal the genotype classification of the field isolates. Besides, Dharmayanti *et al*. [29] have stated the importance of identifying prevalent serotype(s) isolated in a region to investigate the cross-protective potentials using currently available vaccines. In particular, this study attempts to focus on pathogenic 4/91-like and QX-like IBV infections, pioneering a field-based scientific analysis on these two strains that are actively circulating in Indonesian poultry industry.

Thus, the current study may reveal clear molecular characteristics of these two IBV strains as a means to support the implementation of various control strategies, particularly vaccination-related actions. In short, any findings are expected to provide a basis for an effective vaccination strategy in the prevention and control of IBV.

## Materials and Methods

### Ethical approval

Diagnostics samples used in this research were obtained from commercial poultry farms suspected to IBV infection(s). No experimentation on live animals or infection study was undertaken. All diagnostic samples were being conducted in accordance with all applicable laws of the Government of Indonesia.

### Samples and virus isolation

As samples, 10 pools of layer chickens, each of which is mixed from 2-3 individual chickens, taken from various existing flocks in different commercial poultry farms within Central Java and Yogyakarta regions, Indonesia. Among all commercial poultry farms in these regions, at first, those selected were farms and flocks that have been suspected of being infected by IBV. Information on the suspicion was gathered from veterinarians practicing in each commercial poultry farm. Next, layer chickens were carefully selected from those manifesting probed clinical signs such as respiratory problems, “false layer” syndrome, drops in egg production, and reduced egg quality. Besides, potential samples were deducted to those exhibited a “penguin-like” stance. It is surgically proven by applying necropsy on those samples, by which cystic oviduct and swollen as well as reddish kidney were observed. After the deduction, those manifesting all required statistical and physical signs were selected as samples for this study. Then, these samples were taken to the Laboratory of Microbiology, Department of Microbiology, Faculty of Veterinary Medicine, Gajah Mada University (FKH-UGM) for being diagnosed.

To isolate disease-causing agent, samples in the form of cecal tonsil and trachea from the selected chickens were collected in the laboratory. Next, these samples were crushed to be later mixed with phosphate-buffered saline using a vortex device to produce virus suspensions. After that, about 0.2 mL supernatant from sample suspensions was inoculated in *allantoic cavity* of specific pathogen free (SPF) or IBV antibody neutral 10-day-old embryonated eggs. These inoculated eggs were then incubated at 37°C temperature. After being inoculated for 48 h, allantoic fluids were harvested from these incubated eggs. Virus suspensions from both the gathered fluids and the rest of sample supernatant were stored at −78°C temperature for further analyses.

### RNA extraction and polymerase chain reaction (PCR) amplification and sequencing

Viral RNA was extracted from stored tissue supernatant or allantoic fluids using Viral Nucleic Acid Extraction Kit II (Geneaid, New Taipei, Taiwan) according to the manufacturer’s protocol for diagnosis and sequencing. Positive control of virus was Mass strain, originated from a commercial vaccine. Reverse transcriptase (RT)-PCR was conducted using MyTaq™ One-Step RT-PCR Kit (Bioline). Next, amplification on S1 gene fragment was conducted using primer referring to the prior work of Capua *et al*. [32], which had a forward primer: 5’-aca tgg taa ttt ttc aga tgg-3’; reverse primer: 5’-cag att gct tac aac cac c-3’; and PCR product length: 383 bp. A total of 25 µL mixture consisting of 2.5 µL RNA (20-50 ng), 0.25 µL RT, 0.5 µL RiboSafe RNase Inhibitor, 12.5 µL 2x MyTaq One-Step Mix, and 1 µL (200 nm) each of specific forward and reverse primers targeting S1 gene of IBV [32] and RNase-free distilled water was prepared. The reaction conditions were as follows; First, RT was conducted at 42°C for 20 min, which was followed by pre-denaturation at 95°C for 1 min. Next, PCR was conducted for 40 cycles of denaturation at 95°C for 10 s. It was followed by an annealing at 49°C for 10 s and an extension at 72°C for 30 s. Then, a final extension was performed at 72°C for 5 min. Then, PCR product was analyzed with electrophoresis in 2% agarose gel. This RNA extraction until electrophoresis steps were conducted at the Laboratory of Microbiology, Department of Microbiology, FKH-UGM, and then the PCR products were sent to the First BASE (Apical Scientific, Selangor, Malaysia) for being sequenced.

### Sequence alignment and phylogenetic analysis

Nucleotide sequences of S1 gene fragment were assembled and aligned using BioEdit software [33]. A total of 47 IBV S1 reference sequences including Mass, Conn, 4/91, and QX-type vaccine strains were taken from GenBank [34]. They were aligned with sample sequences and cut into the same length (318 bp). Next, FASTA file of the alignment was analyzed for revealing its phylogenetic by applying the neighbor-joining method with 1000 bootstrap replicates on MEGA-X software [35]. Amino acid alignment was constructed by BioEdit software. The amino acid number starts from the first open reading frame of 4/91 (AF093794.1) and QXIBV (KC795604.1) S1 genes.

## Results

### Clinical characteristics and pathological finding

**Figure-1:**
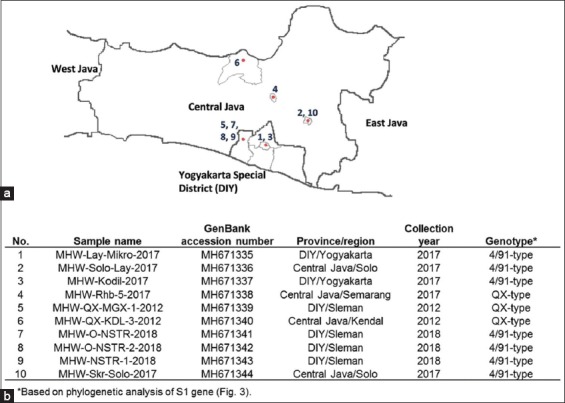
Distribution and identity of infectious bronchitis virus samples. (a) Samples were gathered from Central Java and Yogyakarta regions. Numbers represent samples analyzed in this study. Identity of samples is summarized in (b) and ordered based on accession numbers.

Figure-1a and b show the list of samples selected and used in this study. The periods of sample selection, gathering, and isolation vary following the emergences of suspected IB infection(s) in commercial poultry farms. The periods include 2012 (two samples), 2017 (five samples), and 2018 (three samples). In particular, samples gathered in 2018 (MHW-O-NSTR-2018, MHW-O-NSTR-2-2018, and MHW-NSTR-1-2018) were taken from one commercial poultry farm; however, they were sourced from different flocks on the farm.

**Figure-2:**
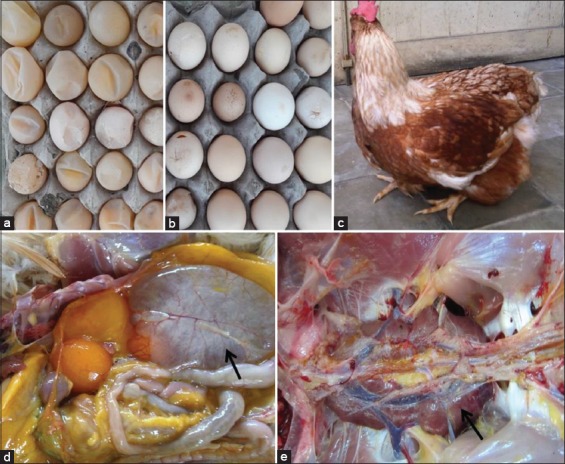
Characteristics of infectious bronchitis virus-infected chicken. Low quality of eggs such as soft (a), thin and breaking shells (b). “Penguin-like” stance or standing with lower abdomen (c) as the built-up internal pressure. At necropsy, enlarged, thin, and transparent oviduct filled with liquid (cystic oviduct) (d) and swollen and hemorrhagic kidney (e) are observed.

All samples were taken from layer chickens that manifest clinical signs of IB disease, including respiratory problems, drops in egg production, and reduced egg quality. Besides, a physical characteristic of “penguin-like” stance or standing with lower abdomen was also reported. During necropsy, an enlarged, thin, and transparent oviduct was discovered. Besides, a swollen and reddish kidney was found (Figure-2). The accumulation of liquid inside the oviduct (cystic oviduct) appears to build up an internal pressure that accounts to “penguin-like” stance. All flocks were stated by veterinarians practicing in each poultry farm to have received IBV vaccinations using inactivated Mass strain.

### Phylogenetic analysis of S1 sequences

To investigate any genetic relationship among IBV strains infecting the samples, a phylogenetic tree was constructed based on S1 sequences that contain 10 field isolates and 47 reference sequences retrieved from GenBank (https://www.ncbi.nlm.nih.gov/genbank/), including vaccine strains such as 4/91, Mass (H52, H120, and Mass 41), Conn, and QX (QXIBV and D388). Due to the lack of published/existing domestic data sequences on IBV, this study does not include reference strain from Indonesia into the phylogenetic analysis.

**Figure-3:**
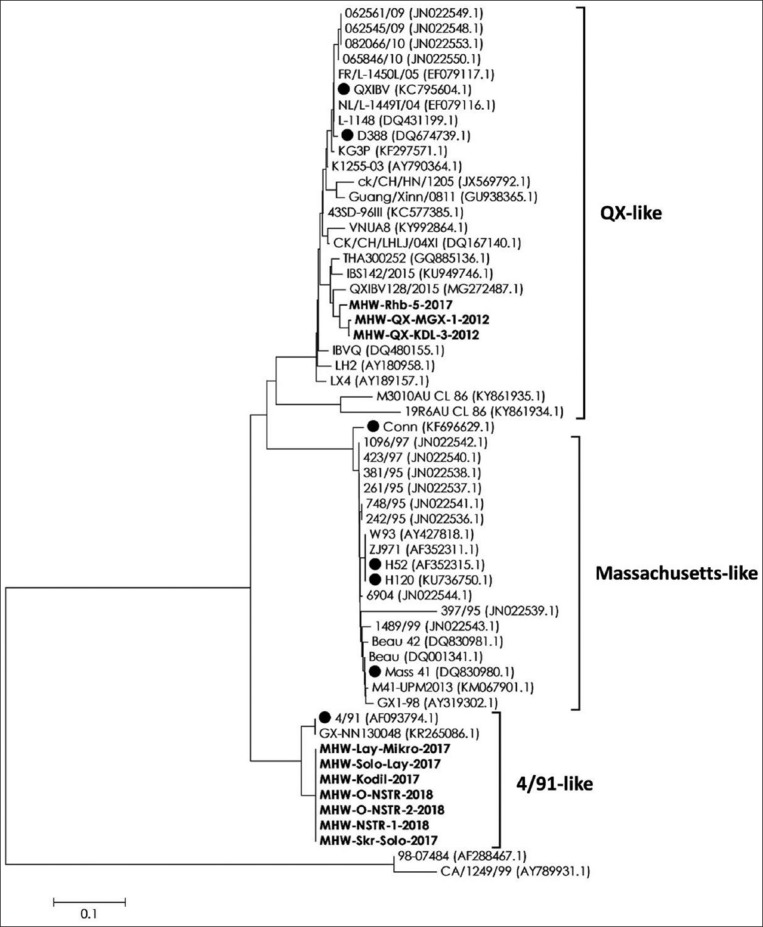
Phylogenetic analysis of S1 gene. Sequence alignment of 318 bp long S1 gene is constructed from 10 field isolates from Central Java and Yogyakarta in conjunction with 47 reference strains. Phylogenetic analysis is constructed using neighbor-joining method with 1000 bootstrap replicates. Sequences in bold are field isolates. The reference vaccine strains are marked with black circle.

**Figure-4:**
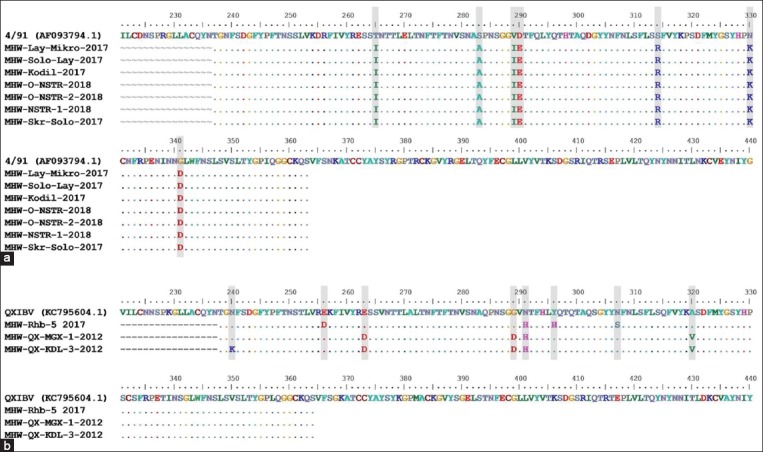
Sequence alignment of 127 amino acid length S1 protein. (a) Alignment of 4/91 strain (AF093794.1) with Indonesia isolates from 4/91-like cluster. (b) Alignment of QX strain (KC795604.1) with Indonesia isolates from QX-like cluster. Position of different amino acids is shaded. Alignment is generated with BioEdit software [33].

Figure-3 shows the results of the analysis, in which there were two distinct clusters that contain IBV isolates from Central Java and Yogyakarta regions, which were 4/91-like and QX-like strains. The genetic relationship from 318 bp S1 gene showed that 7 of 10 isolates (MHW-Lay-Mikro-2017, MHW-Solo-Lay-2017, MHW-Kodil-2017, MHW-O-NSTR-2018, MHW-O-NSTR-2-2018, MHW-NSTR-1-2018, and MHW-Skr-Solo-2017) were grouped in 4/91-like cluster. All these 4/91-like isolates were gathered in 2017 and 2018, in which they were originated from various regions in Central Java and Yogyakarta. Besides, sequence alignment of S1 protein showed that all 4/91-like isolates in Indonesia are homogeneous. When these isolates were compared to 4/91 vaccine strain, there was a pattern of six amino acids conserved in seven positions, including isoleucine (I; residues 265, 289), alanine (A; 283), glutamic acid (E; 290), arginine (R; 314), lysine (K; 330), and aspartic acid (D; 341) (Figure-4a) [33].

On the other hand, 3 of 10 isolates (MHW-Rhb-5-2017, MHW-QX-MGX-1-2012, and MHW-QX-KDL-3-2012) were grouped into QX-like cluster (Figure-3). Based on the periods of isolation, two of them were isolated in 2012, while one was isolated in 2017. All these QX-type isolates were originated from different flocks in different regions (Figure-1b). Besides, sequence alignment of S1 protein showed that all QX-like isolates in Indonesia are considerably heterogeneous. In particular, there are four amino acid positions (residue 263, 289, 291, and 320) that distinguish isolates taken during 2012-2017. In fact, these positions were rather conserved for isolates from 2012 (Figure-4b). Then, among all QX-like isolates in Indonesia, there was a conserved amino acid (histidine) at residue 291 when compared to QX reference vaccine strain.

## Discussion

Circulating IBV strains in Indonesia have been reported to have similarities to globally distributed IB vaccine strains, including Australia-origin N2/62 and US-origin Conn 46 as well as Mass 41 [17,30,31]. However, cases of IB disease in the country are continuously occurring even within actively IB-vaccinated chicken flocks, suggesting insufficient vaccine protection for currently circulating IBV strains on the fields. Meeusen *et al*. [36] have stated vaccination program as the most effective action to control IBV infections; however, it continuously faces challenges and obstacles due to the virtually endless emergence of new IBV serotypes. In the current situation, there are more than 50 identified IBV serotypes, signifying high potentials of less or no cross-protection between vaccines with different basis of IBV strains [16,37,38]. Therefore, a continuous characterization of virus isolates on the fields is critical to design a properly measured strategy to prevent IB disease. For this study, samples are gathered from layer chicken flocks in Central Java and Yogyakarta regions. Flocks experiencing respiratory problems and drops in egg production, while its chickens are showing peculiar signs of “penguin-like” stance, are stated as indications of an IBV infection. During necropsy, nephritis and cystic oviduct were also observed, suggesting the causing agent as a nephropathogenic strain. Looking at these clinical signs and necropsy result, these chickens are stated as experiencing “false layer” syndrome. Srinivasan *et al*. [39] have noted “false layer” syndrome as an explanatory indication of poultry health condition that may show a normal egg production but actually infected. The infection can be confirmed during a necropsy, in which the body cavity of a chicken is filled with fluids or coagulated yolk. Meanwhile, Srinivasan *et al*. [39] have also noted penguin-like stance as probably being caused by progressive accumulation yolk material, partially or fully along with an inflammatory exudate in the abdominal cavity. Then, a discovery of ovarian cyst alongside drops in egg production at chicken samples is also indications of “false layer” syndrome, as suggested by Landman *et al*. [40].

Furthermore, phylogenetic and genetic analyses are two means in monitoring the molecular evolution of IBV and in delivering proven and accurate evidence on its genotype classification [41]. In this study, all samples are obtained from chickens with similar signs; however, a phylogenetic analysis of their S1 gene fragment reveals two distinctive IBV genotype clusters, which are stated as 4/91-like and QX-like. Isolates included in the 4/91-like cluster are MHW-Lay-Mikro-2017, MHW-Solo-Lay-2017, MHW-Kodil-2017, MHW-Skr-Solo-2017, MHW-O-NSTR-2018, MHW-O-NSTR-2-2018, and MHW-NSTR-1-2018. In other words, the current study reveals 4/91-like cluster to account for 7 of 10 isolates under investigation. As a matter of facts, Bande *et al*. [22] have reported that, despite the restriction of several IBV genotypes to certain geographic region(s), several strains such as Mass, IBV 4/91, and QX-like IBV are more global in terms of their distributions. Basically, 4/91 reference is a vaccine and pathogenic strain originated from Europe, from which the 4/91 IBV strain has spread worldwide.

Furthermore, phylogenetic analysis and sequence alignment showed that all 4/91-like isolates gathered from commercial poultry farms in Indonesia are genetically homogeneous. However, there are differences in terms of amino acids compared to 4/91-reference vaccine strain. In other words, the finding indicates that the circulating 4/91-like isolates are not originated from vaccine strains but from pathogenic strains on the fields. In fact, several reports have suggested various IBV infections due to 4/91 strain, including Mahdavi *et al*. [42] who have proven IBV infections due to 4/91 strain to cause a broad tissue distribution at respiratory, digestive, and urinary tract tissues. The virus strain is a nephropathogenic IB virus that has higher pathogenicity on the kidney compared to other organs. Besides, Amjad *et al*. [43] have found 4/91 IBV strain to be more enterotropic, which is indicated by its prolonged persistence at cecal tonsil compared to trachea. Similar view has also been suggested by Dhinakar and Jones [44] who have reported 4/91 IBV strain to be more enterotropic than pneumotropic.

In the current literature, 4/91 strain has been widely reported, in which the strain has been largely associated with nephritis; however, it is not described to develop cystic oviduct [17,45]. On the other hand, Landman *et al*. [40] have reported cystic oviduct at active ovaries to be found in chickens infected by IBV, particularly QX strain. It is parallel with a study of Cook *et al*. [1] and Abro *et al*. [46] who have noted IBV infections due to Europe-origin QX-like strain to be associated with nephropathogenicity and cystic oviduct. Interestingly, this study discovers typical QX symptoms, including nephritis and cystic oviduct, in chickens at which 4/91-type IBV was isolated. In other words, it suggests that clinical manifestations of the disease may not purely reflect genotypes of IBV strain. The fact has been supported by a prior study in Hungary which has reported the detection of QX and 4/91 strains in poultry farms without any noticeable clinical signs and drops in egg production [47].

Meanwhile, a cocirculation of 4/91, QX, and Mass strains in Belgium has been reported to occur in poultry farms experiencing similar respiratory signs and egg deformity without any reported nephritis and cystic oviduct [48]. In the literature, many factors have been suggested to increase exposure to an infection on chickens, including genetic line, dietary protein source, chilling, and other stress factors [17]. A molecular identification is, hence, necessary to differentiate the causing strains [12,17,49].

Looking at the results of this study, the QX-like cluster consists of three isolates (MHW-QX-MGX-1-2012, MHW-QX-KDL-3-2012, and MHW-Rhb-5-2017). Samples, at which strains grouped in the cluster are found, were collected from three different flocks in Central Java and Yogyakarta. Their collection periods apparently differ (2012 and 2017), and hence, it suggests that these QX-like strains have been circulating since 2012 within these regions. Basically, QX reference strain has been described as nephropathogenic although it is initially linked with proventriculitis [18,19]. Apparently, QX-like isolates in Indonesia have amino acid variations compared to the reference QX vaccine strains. It indicates that isolates in the country observed by this study are considered as pathogenic strains that have been circulating on the fields. However, sequence alignment shows that isolates from 2012 (MHW-QX-MGX-1-2012 and MHW-QX-KDL-3-2012) have four amino acid differences compared to those of QX-like isolate from 2017 (MHW-Rhb-5-2017). It may have suggested a possible mutation occurring before 2012 which results in QX-like IBV strains.

In contrast, the amino acid differences may appear as a logic result because the target of amplification in this research is S1 gene fragment, which is included among the first 395 amino acid classified as HVRs [50]. The genetic variations are, hence, possible to emerge as spontaneous mistakes (nucleotide substitution, deletion, insertion, or recombination) during viral replications [17]. In this study, strains classified into cluster QX-like are recognized as causing chickens to show physical and statistical signs such as drops in egg production, penguin-like stance, and thin as well as rough eggshell. Besides, swollen kidney and cystic oviduct are discovered during necropsy. These conditions are parallel to the work of Landman *et al*. [40] who discovered infections on kidney and oviduct in QX-like-infected SPF as well as commercial chickens. Infections on the kidney caused by QX-related strains have also been reported by Gough *et al*. [51]. Meanwhile, Benyeda *et al*. [24] have discovered varied clinical signs and pathological changes of strains despite close genetic relationships between all isolated QX-like strains. Besides, mortality and morbidity are varied among infected poultry. It is widely found that infections due to QX-like strains have been causing respiratory lesions, nephroso-nephritis, oviduct dilatation, and ileum lesions.

Another interesting finding in this study is the occurrence of “false layer” syndrome at IBV-infected chickens due to strains classified into 4/91-like cluster. In fact, it is contrast to the findings of Landman *et al*. [40] and Benyeda *et al*. [24], who have stated QX-variants of IBV as the disease-causing agent of respiratory problems, nephritis, drops in egg production, and “false layer” syndrome in infected chickens. Primarily, Crinion and Hofstad [52] have suggested “false layer” syndrome to possibly occur in chickens infected by certain IBV strains in their early ages. Meanwhile, its infections in layer chickens have been causing various indications, including pigmented eggshell and drops in egg production [53]. In particular, only a few IBV strains have been reported to cause “false layer” syndrome [24,54]. The syndrome is induced through infections in young chickens by various strains of IB virus, including Mass, Australian T, and several QX strains [24,53]. Meanwhile, the current literature has never formally reported 4/91 strains as a causing agent of “false layer” syndrome. Due to its huge risks to the sustainability of commercial poultry industry, “false layer” syndrome must be prevented through active and early protection to IBV infection(s).

Then, samples collected and used in this study, as aforementioned, were sourced from commercial poultry farms that have actively applied vaccination protocols using inactivated Mass strain. Nevertheless, IBV infections have persistently been causing problems in those farms. Molecular analyses in this study have discovered IBV-infected samples of layer chickens in Indonesia to be included in 4/91 or QX clusters. In other words, currently applied vaccines based on Mass strain are indeed protective as no sample is discovered to be IBV infected by Mass strain. However, this type of vaccine is clearly inadequate to give protection against 4/91 and QX strains. Mass serotype is the most widely used strain for vaccination protocols all over the world. Gaba *et al*. [55], in fact, have stated the excessive use of Mass strain in various IBV cases as a critical cause of very poor cross-protection against 4/91 IBV strains. Hence, it significantly increases potential failures of IBV vaccination programs. In the work of Parson *et al*. [56], IBV vaccines based on Mass strain have also been proven as being inefficient in delivering protections against 4/91 IBV strain. Virus IB itself has been acknowledged for its high mutation, by which a minor change in S1 sequence would swiftly cause poor cross-protection and vaccine failure. Cavanagh [57] has reported that as low as 5% differences in S1 sequence would produce a poor cross-protection, which would surely increase the potential of IBV infections despite active vaccination protocols applied in commercial poultry farms. Basically, vaccine(s) based on a certain serotype or genotype would be able to protect well-vaccinated chickens against a homologous challenge [58]. Homologous vaccine strain is, hence, stated as having the best delivery of protection against a certain infection; however, partial protection is possible to achieve through an appropriate and careful combination of heterologous vaccines [59]. It has been widely recognized that a vaccination protocol that applies two antigenically distinct LAV, for example, Mass and 4/91, can result in a broad cross-protection against many different IBV types [19,60,61]. Therefore, a new vaccination program that delivers cross-protection against multiple IBV strains is clinically required. Results of this study also indicate all IBV isolates under investigation as new pathogenic 4/91 and QX strains circulating on the fields. There are, however, limitations of the current work, including a limited length of S1 gene being observed (383 bp) which cannot reflect the whole molecular characteristics of the virus. Completing the genotyping and serotype analysis of IBV infecting those isolates will, hence, be delivering more comprehensive data of this new pathogenic IBV circulating in Indonesia.

## Conclusion

This study has successfully conducted a molecular analysis on disease-causing virus in isolates from commercial poultry farms in Indonesia showing clinical signs of IB disease. After the molecular analysis has been conducted by amplifying the S1 fragment gene of IBV isolates, this study discovers new pathogenic 4/91-like and QX-like IBV strains circulating in the country. In fact, this is the first molecularly proof report on these two strains in Indonesia. In general, this study reveals the infection of 4/91-like strain to exhibit signs that resemble a QX-like infection such as nephritis and cystic oviduct. Besides, “false layer” syndrome is discovered on all samples infected by 4/91-like IBV strain. As previous studies have investigated the low cross-protection capability of existing IBV vaccines to strains circulating in Indonesia, this study confirms the above-mentioned strains to have different variances than existing vaccines. In other words, this study suggests molecular characteristics of the two strains as a basis for the selection of virus strains in the making of proper IBV vaccines applied to commercial poultry farms in Indonesia. Furthermore, results of this study may also be used in establishing a more effective vaccination strategy for the country’s poultry industry as a means to control the circulation and spreading of IB disease. For example, the enhanced strategy may include a combination of heterologous vaccines. Besides, it may include the application of a newly developed vaccine that is clinically fitted with IBV virus strain(s) circulating on the fields. According to previous reports, IBV has been recognized to have a high mutation rate; therefore, future researches may focus on active and periodic monitoring on IBV strains circulating and mutating in Indonesia to support the country’s control and preventive programs of IB disease. Then, the discovery of 4/91-like and QX-like clusters has brought up a further need to check their real pathogenicity. As the current study is intended to focus on molecular analysis of IBV strains that manifest certain clinical signs in infected chickens, a challenge experiment on SPF chickens is, thus, required to prove the pathogenicity of the discovered IBV strain clusters.

## Authors’ Contributions

MHW devised the project, conceived the research design, handled collaborative works with external partners, collected samples, and performed laboratory as well as experiments. TEG performed computational preparations and led the writing of first manuscript draft in consultation with MHW and WA. WA verified laboratory and data analysis methods. MHW, TEG, and WA analyzed and interpreted the data and results of experiments. MHW worked out all revisions in consultation with WA and TEG. MHW conducted manuscript revision. All authors read and approved the final manuscript.
